# Functional characterization and immunomodulatory properties of *Lactobacillus helveticus* strains isolated from Italian hard cheeses

**DOI:** 10.1371/journal.pone.0245903

**Published:** 2021-01-25

**Authors:** Miriam Zago, Lucia Massimiliano, Barbara Bonvini, Giuseppe Penna, Giorgio Giraffa, Maria Rescigno

**Affiliations:** 1 CREA Research Centre for Animal Production and Aquaculture (CREA-ZA), Lodi, Italy; 2 Department of Experimental Oncology, European Institute of Oncology, Milan, Italy; 3 Mucosal Immunology and Microbiota Unit, Humanitas Clinical and Research Center, IRCCS, Rozzano (Mi), Italy; 4 Department of Biomedical Sciences, Humanitas University, Pieve Emanuele (Mi), Italy; Institute of Molecular Genetics and Genetic Engineering, SERBIA

## Abstract

*Lactobacillus helveticus* carries many properties such as the ability to survive gastrointestinal transit, modulate the host immune response, accumulate biopeptides in milk, and adhere to the epithelial cells that could contribute to improving host health. In this study, the applicability as functional cultures of four *L*. *helveticus* strains isolated from Italian hard cheeses was investigated. A preliminary strain characterization showed that the ability to produce folate was generally low while antioxidant, proteolytic, peptidase, and β-galactosidase activities resulted high, although very variable, between strains. When stimulated moDCs were incubated in the presence of live cells, a dose-dependent release of both the pro-inflammatory cytokine IL-12p70 and the anti-inflammatory cytokine IL-10, was shown for all the four strains. In the presence of cell-free culture supernatants (postbiotics), a dose-dependent, decrease of IL-12p70 and an increase of IL-10 was generally observed. The immunomodulatory effect took place also in Caciotta-like cheese made with strains SIM12 and SIS16 as bifunctional (i.e., immunomodulant and acidifying) starter cultures, thus confirming tests in culture media. Given that the growth of bacteria in the cheese was not necessary (they were killed by pasteurization), the results indicated that some constituents of non-viable bacteria had immunomodulatory properties. This study adds additional evidence for the positive role of *L*. *helveticus* on human health and suggests cheese as a suitable food for delivering candidate strains and modulating their anti-inflammatory properties.

## Introduction

Lactic acid bacteria (LAB) are so far the main used microorganisms in functional foods, which can be defined as natural or processed foods that contain biologically active compounds which provide documented health benefits [1]. Within functional foods are included probiotics, which exert beneficial effects on the health state through two essential mechanisms: a direct action by live microbial cells (or even dead but with intact cell wall, defined in this case as ‘paraprobiotics’), and an indirect effect, generally through fermentation, which generates a wide range of metabolites, such as bioactive peptides, exopolysaccharides, bacteriocins and organic acids produced during the fermentation process, collectively defined as ‘postbiotics’, an evolving term in the field of functional foods [2–9]. *Bifidobacterium bifidum*, *Bifidobacterium breve*, *Lactobacillus acidophilus*, *Lactobacillus gasseri*, *Limosilactobacillus* (*Lim*.) *reuteri*, *Lim*. *fermentum*, *Lactobacillus johnsonii*, *Lacticaseibacillus (Lact*.*) (para)casei*, *Lact*. *plantarum*, and *Lact*. *rhamnosus* are, among others, the LAB species prevalently used in the formulation of probiotic or functional cultures [10]. However, some emerging LAB species, such as *Lactobacillus helveticus*, are gaining increasing attention of the food industry, especially in the dairy sector.

*Lactobacillus helveticus* is a multifunctional thermophilic LAB with a generally recognized as safe (GRAS) *status* and, undoubtedly, one of the most promising probiotic LAB species. It shows a range of pro-technological features, such as the ability to produce large quantities of lactic acid in milk and release, after autolysis, a complex series of enzymes in the matrix, be it fermented milk or cheese, which make it particularly suitable for a dairy application [11]. Moreover, *L*. *helveticus* carries many properties such as the ability to survive gastrointestinal transit, modulate the host immune response, accumulate biopeptides in milk, and adhere to the epithelial cells that could contribute to improving host health [12, 13]. Not less important are the indirect benefits brought by this bacterium to the human host in terms of improving the bioavailability of nutrients and removing food allergens and other unwanted molecules [7]. *In vivo* studies in murine models also showed that *L*. *helveticus* may prevent gastrointestinal infections, enhance protection against pathogens, and positively affect the composition of the intestinal microbiota [14–16]. Interestingly, comparative genomics studies have shown a phylogenetic affinity and a remarkable similarity in gene content, especially for key gene sets that facilitate adaptation to food matrices or the gastrointestinal tract, between *L*. *helveticus* and many intestinal lactobacilli, including *L*. *acidophilus* [13, 17]. *Lactobacillus helveticus* could be then a valuable source of candidate strains to design new functional foods.

The purpose of this work was to investigate the applicability as functional cultures of four *L*. *helveticus* strains isolated from cheese. The strains, which showed *in vitro* functional traits, were tested as starters in experimental soft cheeses to verify their persistence in cheese-making conditions. This study adds further evidence on the potential of *L*. *helveticus* on human health and suggests cheese as a suitable substrate to vehiculate candidate strains.

## Materials and methods

### Bacterial strains

*Lactobacillus helveticus* strains SIM12, SIS16, 1734 and Lh43 isolated from Italian hard cheeses were included in this study. The strains were reactivated overnight at 37°C in MRS (De Mann Rogosa and Sharpe) broth (Merck, Darmstadt, Germany, catalog number 1.10661.0500). *Lact*. *rhamnosus* ATCC 7469, used as auxotrophic strains in folate production analysis, was grown overnight at 37°C in MRS (Merck). *Salmonella enterica* serovar *typhimurium* strain SL1344 (FB62) was provided by G. Dougan (The Welcome Trust Sanger Institute, Hinxton, UK) and grown in LB (Luria-Bertani) broth (Sigma, Milan, Italy, catalog number L3022). *L*. *helveticus* strains were grown to an OD600 = 0.6 in MRS medium at 42°C. For the experiments with human peripheral blood mononuclear and dendritic cells cultures were centrifuged and pelleted cells were washed twice in IMDM (Iscove’s Modified Dulbecco’s Medium) (Sigma, St. Louis, USA, catalog number I3390) broth to eliminate residual MRS medium. Washed cells were then incubated at 37°C for 18 h in IMDM. Strain FB62 was grown at 37°C in LB medium (Sigma). The obtained *L*. *helveticus* cultures were used as such or filter sterilized (0.45 μm, Merck Life Science, Milan, Italy). Cell-free culture supernatants were called ‘postbiotics’.

### Folate production

*Lactobacillus helveticus* strains were screened for the ability to produce folate. After overnight growth at 32°C in MRS, re-activated cultures were washed three times with a NaCl solution (0.85% w/v). Resuspended cells were used to inoculate (2%; v/v) a folate-free medium (Folic Acid Casei Medium, Difco, Sparks, Maryland, USA). Cultures able to grow in the folate-free mediaum were sub-cultured three times in the same medium before the determination of folate concentration in the supernatants (ng/ml). Tests were carried out in triplicate by a microplate microbiological assay according to Laino *et al*. [17], using *Lacticaseibacillus rhamnosus* ATCC 7469 as auxotrophic strain in folate determination, as it can growth only in presence of folate. Briefly, cells from an overnight culture of the indicator strain were harvested by centrifugation, washed with 0.85% of NaCl, inoculated (2% v/v) in the folate-free medium, and loaded into 96-wells microplates. A calibration curve was performed, adding folic acid (Merck, Darmstadt, Germany) standard solution to each inoculated well. Filter-sterilized (0.22 mm) culture supernatants were diluted with the extraction buffer and added to each inoculated well. Both standards and extracted samples were loaded in triplicate. Microplates were incubated for 48 h at 32° C and the OD650 was scored. Total folates were quantified according to the cubic polynomial function given by the standard curve. The values obtained were expressed as ng/ml.

### Antioxidant activity in milk

After overnight growth at 42°C in MRS broth, the lactobacilli were sub-cultured twice in sterile, reconstituted skimmed milk (10% w/v) to prevent any interference of the MRS medium in the determination of the antioxidant activity (AO). After 48–72 hours of incubation, the coagulated cultures were centrifuged and the supernatants used for the determination of AO, which was carried out by measuring the radical scavenging activity, the ferric reduction capacity (FRAP), and the accumulation of free thiol groups according to Perna *et al*. [18]. The radical scavenging activity was carried out by the ABTS (2,2’-azino-bis-3-ethylbenzothiazoline-6-sulfonic acid) method and expressed as percentage inhibition (I%) of the ABTS·+ radical, whereas FRAP and free thiol groups values were expressed as μM Fe(II) and μM-SH, respectively. The cultures in reconstituted skimmed milk were carried out in triplicate and the values were averaged.

### Enzymatic activities

The o-nitrophenyl-ß-D-galactopyranoside (Sigma-Aldrich, Steinheim, Germany) substrate was used to determine the ß-galactosidase activity as described by Vinderola and Reinheimer [19]. The proteolytic activity in sterile skimmed milk was evaluated by the o-phthaldialdehyde (OPA) method [20]. Briefly, after incubation of the strains in sterile skimmed milk at 37°C for 48 h, proteins in 1 ml milk culture samples were precipitated. After centrifugation and filtration of the resulting supernatants, the o-phthaldialdehyde reagent (Sigma-Aldrich, Steinheim, Germany) was added and the proteolytic activity was measured by OD reading at 340 nm (Jasco, Great Dunmow, UK). The results, which were the average of triplicate samples, were expressed as μg/ml of α-amino groups released in the medium. Glycine (Fluka, Steinheim, Germany) was used as standard to build the calibration curve (concentration range 0–75 μg/ml). Aminopeptidase activity was evaluated as described by Gatti *et al*. [21]. Briefly, cells were resuspended at an OD_550_ among 1.2–1.5 and incubated with 0.656 mM/l solutions of Phe-Pro-βNa, Arg-βNa, Lys-βNa, Leu-βNa and Pro-βNa substrates (Bachem Feinchemikalien AG, Bubendorf, Switzerland) for 30 min at 40°C. The activities were evaluated after OD_580_ reading and results expressed as μM/l β-naphthylamine/ml culture/h. The determinations were performed in duplicate in independent experiments.

### Cheese production and sampling

Strains SIM12, SIS16, Lh43 and 1734 were evaluated for their acidification performance, which was tested during growth in pasteurized microfiltered whole milk (PM milk). MRS overnight growth cultures of the four strains were used to inoculate (1% v/v) 100 ml of PM milk. After incubation at 40°C, pH values were measured by a portable pH meter (Portavo 907, Knick, Zofingen, Switzerland). Strains able to reach a target pH value of 5.3 within 4–6 h were selected as starter cultures to produce Caciotta-like soft cheeses. Cheeses were obtained according to Tidona *et al*. [22] with some modifications. Briefly, 3 l of pre-warmed (40°C) PM milk was inoculated with a single strain (1% v/v), previously grown overnight in the same substrate, and maintained at 40°C until pH decreased to a set value of 6.5. Then, 0.3 ml/l of liquid calf rennet (strength 130,000 International Milk Clotting Units, IMCU/kg) was added. After milk clotting, which occurred in approx. 15 min., the curd was cut, extracted and molded. Cheeses were left in a warm (30°C) room to let the whey to drain until the pH dropped to 5.3, brine salted, moved to a cold room and kept at 4°C for 24 h. Milk acidified at pH 5.3 with 85% (w/v) commercial lactic acid solution (Merck Life Science, Milan, Italy) was used as control (CNTR). Cheese suspensions (CS) were obtained by homogenizing cheese samples (10 g) in 90 ml of sterile 2% (p/vol) trisodium citrate buffer (pH 7.5) through a Stomacher 400 Circulator (PBI, Milan, Italy). Both milk cultures and cheeses were carried out in duplicate.

### Experiments with human PBMC and dendritic cells

Human peripheral blood mononuclear cells (PBMC) were isolated from buffy coats of healthy donors after informed consent, according to the institutional guidelines and approved by the institutional Ethical Committee of the Humanitas Clinical and Research Center (approval date 28^th^ January 2016). Monocytes-derived DC (moDCs) were derived from human peripheral blood monocytes selected with anti-CD14 antibodies coupled to magnetic beads (Miltenyi Biotec, Bologna, Italy). Positive selected CD14^+^ cells were incubated for 6–7 days in complete medium containing 1000 U/ml granulocyte-macrophage colony stimulating factor and 400 U/ml interleukin-4 (Miltenyi Biotec) to obtain immature moDCs. MoDCs were stimulated with strain FB62 (MOI bacteria:moDCs = 10:1) or 10 ng/ml lipopolysaccharides (LPS) (Sigma-Aldrich, USA) and incubated in the presence of bacterial cell cultures (or their postbiotics, prepared as described above) of strains SIM12, SIS16, Lh43, and 1734 cultivated in IMDM medium. Stimulated moDCs without bacterial cells or postbiotics were used as controls. Bacterial cells were also incubated with non-stimulated moDCs. In other experiments, total PBMCs were incubated in the presence of CS of both CNTR (see above) and experimental cheeses, i.e., cheeses fermented by strain SIM12 and SIS16. The CS of experimental cheeses were used directly (TQ-CS) or after pasteurization at 63°C for 30 min (P-CS). Viable cells in TQ-CS and P-CS samples were enumerated in MRS agar. After 1 h incubation and extensive wash, gentamicin (100 mg/ml) was added. After 24 h incubation, the amount of accumulated IL-12p70 heterodimer (moDC) or IL-12p40 chain (PBMC) and IL-10 cytokines was determined by ELISA according to the manufacturer’s instruction (R&D Systems).

### Statistical analyses

**Fig 1:**
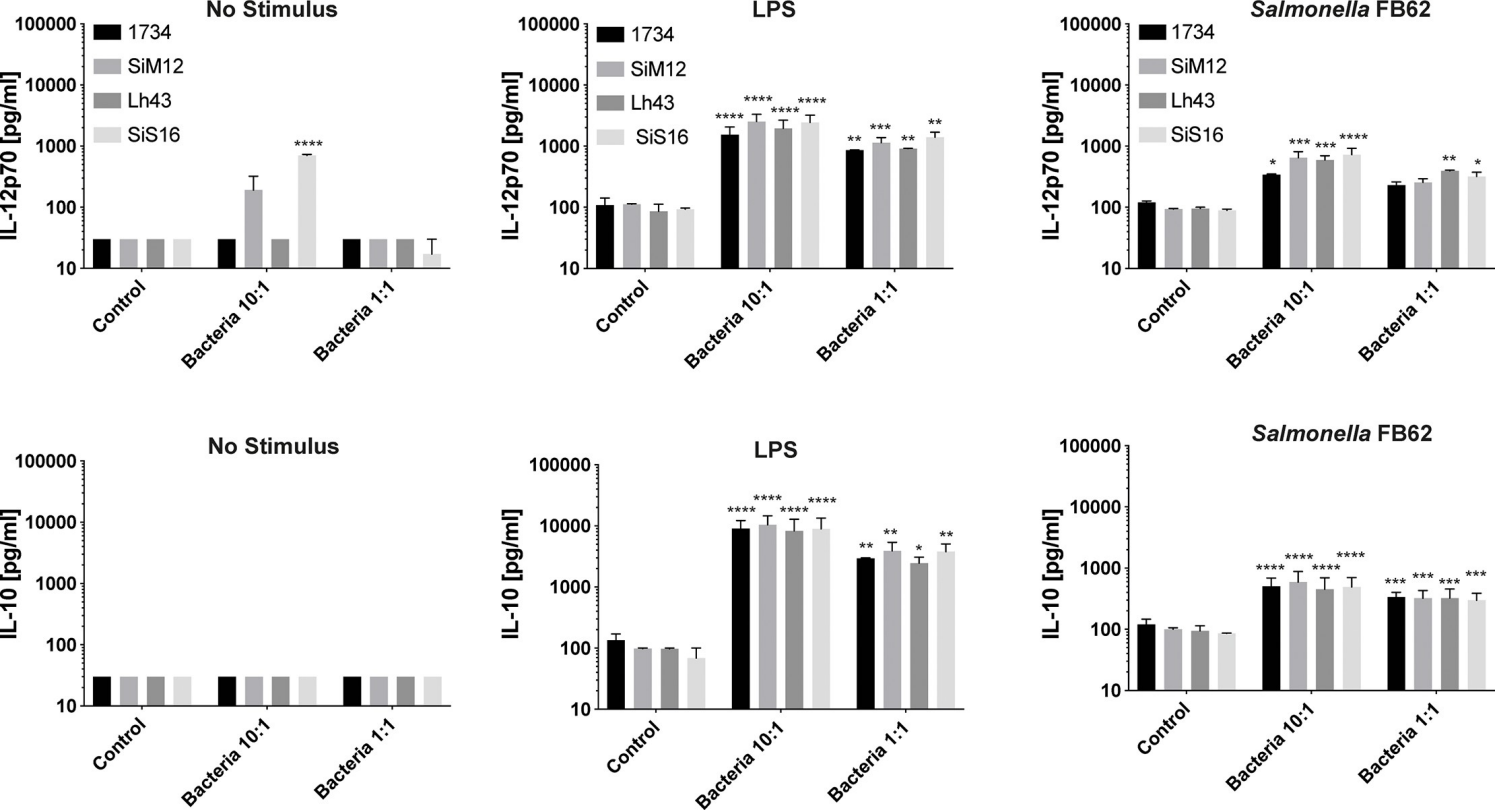
Production of IL-12p70 and IL-10 cytokines in co-culture experiments between moDCs and bacterial cells. The production of IL-12p70 (a, b, c) and IL-10 (d, e, f) cytokines, in co-culture experiments between moDCs and bacterial cells of *Lactobacillus helveticus* SIM12, Lh43, SIS16, 1734 in the presence of lipopolysaccharides (LPS) (b, e), *Salmonella* FB62 (c, f) or in absence of stimulus (a, d) were displayed. Controls: non stimulated (panels a, d) or stimulated (panels b, c, e, f) moDCs without bacterial cells. Statistical significance of single bacteria strain was evaluated by using two-way ANOVA followed by multiple comparisons (Tukey) against the control. Values represent the means ± SD, 95% CI, (*P<0.05, **P<0.01, ***P<0.001, ****P<0.0001).

**Fig 2:**
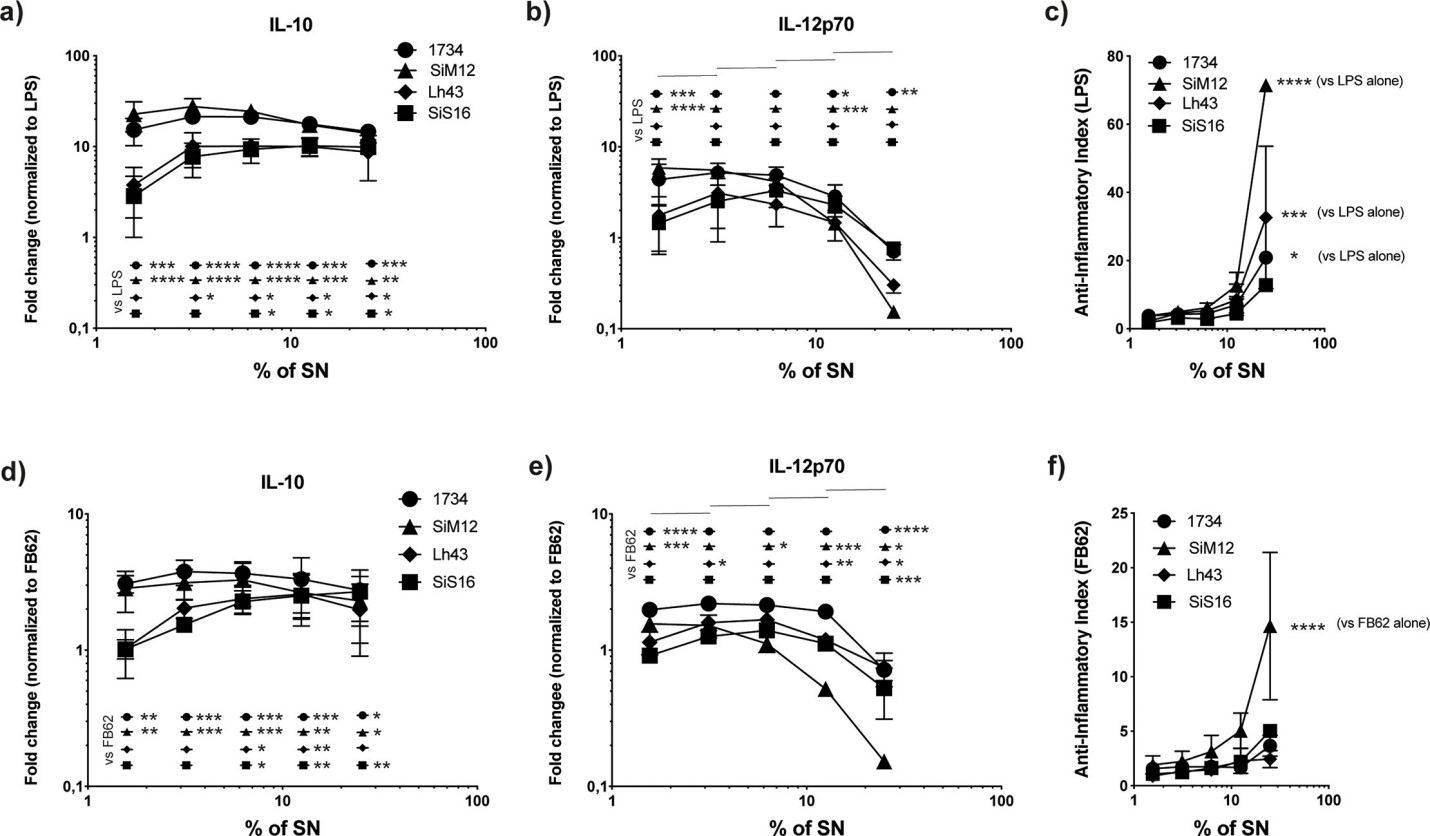
Production of IL-10 and IL-12p70 cytokines in co-culture experiments between moDCs and LPS and *Salmonella* strain in presence of postbiotics. The production of IL-10 and IL-12p70 cytokines, in co-culture experiments between moDCs and lipopolysaccharides (LPS) (a, b) or *Salmonella* FB62 (d, e) in presence of postbiotics (expressed as % added supernatants, % SN -25%, 12,5%,6,25%, 3,12%, 1,56% -) of *Lactobacillus helveticus* SIM12, Lh43, SIS16, and 1734, were displayed. The anti-inflammatory index (IL-10/IL-12p70) is reported for each strain (c, f). Statistical significance of single bacteria strain was evaluated by using two-way ANOVA followed by multiple comparisons (Tukey) against the control and the serial dilution values (b, e). Values represent the means ± SD, 95% CI, (*P<0.05, **P<0.01, ***P<0.001, ****P<0.0001).

**Fig 3:**
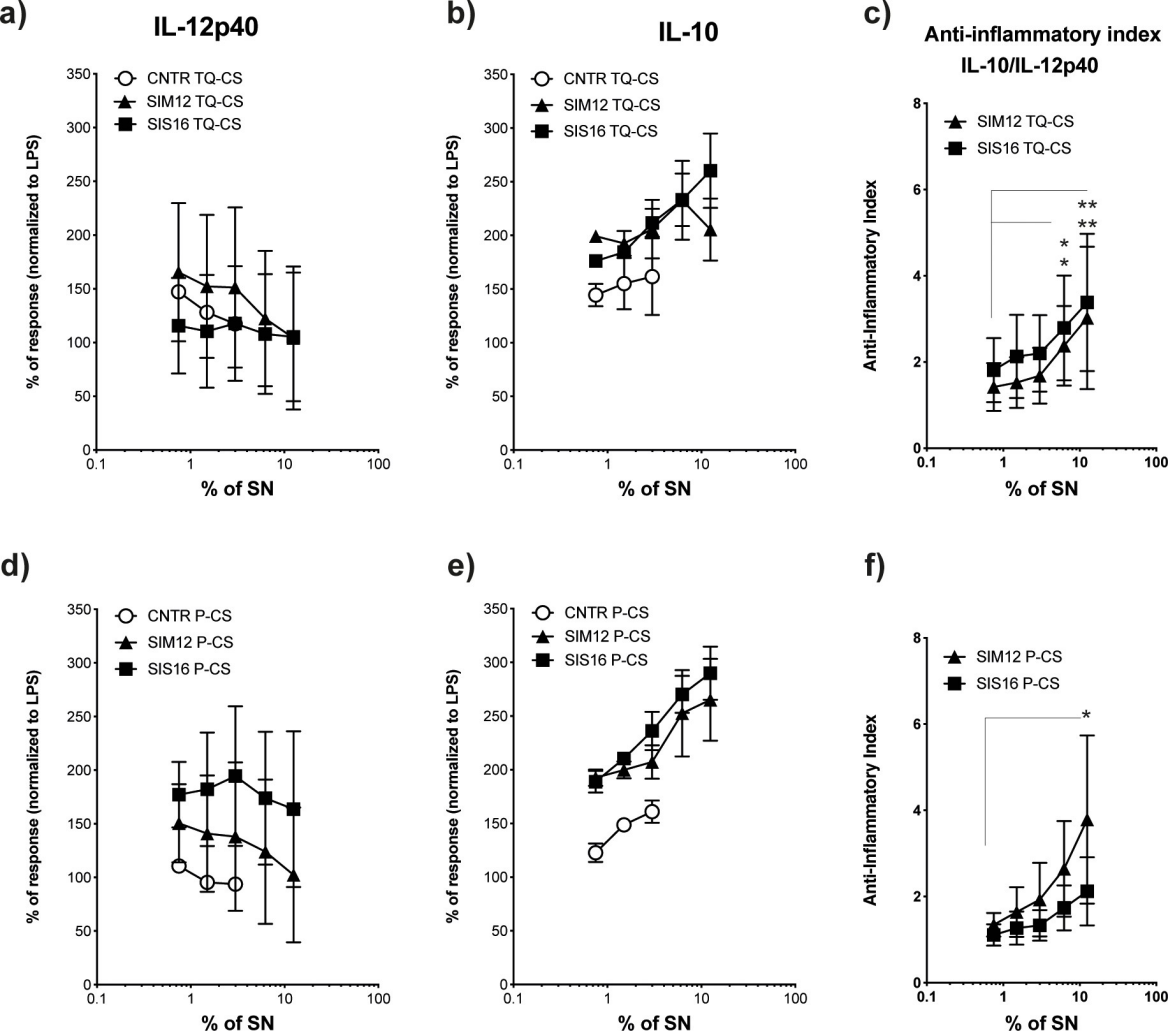
Production of IL-12p40 and IL-10 cytokines in co-culture experiments between PBMCs and LPS in presence of homogenized cheese or homogenized and pasteurized cheese. The production of IL-12p40 (panels a, d) and IL-10 (panels b, e) cytokines in co-culture experiments between PBMCs and lipopolysaccharides (LPS) in presence of homogenized cheese (TQ-CS), or homogenized and pasteurized cheese (P-CS) inoculated with *Lactobacillus helveticus* strains SIM12 and SIS16 and expressed as % of added supernatants (% SN—12,5%, 6,25%, 3,12%, 1,56%, 0,78% -) were showed. Unfermented milk samples acidified at pH 5.3 with lactic acid were used as controls (CNTR). The anti-inflammatory index (IL-10/IL-12p40, panels c, f) is indicated. Statistical significance of single bacteria strain was evaluated by using two-way ANOVA followed by multiple comparisons (Tukey) against the control. Values represent the means ± SD, 95% CI, (*P<0.05, **P<0.01).

Statistical analyses for all figures (Figs 1–3) were performed using GraphPad Prism version 8 for macOS (La Jolla California USA). The comparison of multiple groups was carried out by two-way ANOVA, followed by Bonferroni’s or Tukey’s post-test for correction of multiple comparisons as indicated in figures legend. The data probability value of *P < 0.05 was considered significant.

## Results

### Strain characterization

Results on strain characterization are shown in Table 1. Only SIM12 showed a detectable production of folate, with an amount up to 19.25 ng/ml. The four strains showed a very variable AO activity. SIS16 and SIM12 showed the highest values of FRAP (189 and 216 μM Fe (II) respectively), while SIS16 and LH43 the highest values of ABTS (268 and 489 μM Trolox, respectively). The proteolytic and aminopeptidase activities resulted also very variable. SIS16 and SLh43 showed he highest proteolytic activity (920.50 and 858.82 μg/ml α-amino groups, respectively), whereas the activities on Phe-Pro-βNa, Arg-βNa, Lys-βNa, and Leu-βNa substrates ranged between 0.31–1.61, 0.14–1.19, 0.23–0.46, and 0.73–2.09 μmol/ml*h, respectively. Beta-galactosidase activity ranged from 654.40 (strain SIS16) to >1800 Miller units. Strains SIM12 and Lh43 showed the highest activities, with values >1200 Miller units.

**Table 1:** Functional and technological properties of the *Lactobacillus helveticus* strains investigated.

Strain number	Folate production	Antioxidant activity				
	ng/ml	SH (μM R-SH)		FRAP (μM Fe(II))		ABTS (μM trolox)
1734	<0.4	19		0		0
SIM12	19.25	0		216		122
SIS16	<0.4	0		189		268
Lh43	<0.4	90		70		489
Strain number	Proteolytic activity	Aminopeptidase activity (μmol/ml*h)				β-galactosidase activity
	μg/ml α-amino groups	Phe-Pro-βNa	Arg- βNa	Lys- βNa	Leu- βNa	ng/ml
1734	24.31	1.61	0.90	0.46	2.09	824.26
SIM12	218.81	0.31	0.14	0.23	0.73	>1800
SIS16	920.50	1.03	0.17	0.24	0.78	654.40
Lh43	858.82	1.38	1.19	0.33	0.87	1246.14

SH, thiol groups; FRAP, ferric reduction capacity; ABTS, 2,2’-azino-bis-3-ethylbenzothiazoline-6-sulfonic acid.

Phe-Pro-βNa, phenylalanylproline β-naphthylamide; Arg- βNa, arginine β-naphthylamide; Lys- βNa, lysine β-naphthylamide; Leu- βNa, leucine β-naphthylamide.

### Cytokine production in culture media

When moDCs were stimulated by LPS (or *Salmonella* FB62) and incubated in the presence of *L*. *helveticus* cells, an increased release of both IL-12p70, a pro-inflammatory cytokine which acts as a bridge between innate immunity and adaptive Th1 type immune response (Fig 1, panels b, c), and the anti-inflammatory cytokine IL-10 was retrieved (Fig 1, panels e, f). The production of IL-10 was approx. 10-times higher than that of IL-12p70 in LPS-stimulated moDCs. A dose-dependent effect was observed for all the bacterial strains: the smaller was the ratio bacteria:moDC, the lower was the cytokine production. In the absence of stimulus, strain SIM12 and, especially SIS16, induced a detectable production of IL12p70 with bacteria:cells ratio of 10:1, while a very low production of IL-10 was detected (Fig 1, panels a, d). Fig 2 shows the production of IL-12p70 and IL-10 cytokines (expressed as fold change normalized to LPS and FB62) when moDCs stimulated by LPS or *Salmonella* FB62 were treated with different percentages of culture supernatants (postbiotics) of strains SIM12, Lh43, SIS16, and 1734. Postbiotics showed a dose-dependent, and anti-inflammatory effect on both LPS- (Fig 2, panels a, b) and *Salmonella* driven (Fig 2, panels d, e) inflammatory response, by inhibiting the production of IL-12p70, while strongly increasing the release of IL-10. Postbiotics from all the strains showed different potency in the modulation of IL-12p70 and IL-10 release with SIM12 and Lh43 having the highest anti-inflammatory index (Fig 2, panels c, f).

### Cytokine production in cheese

Although all four strains showed *in vitro* immunomodulatory activity, their ability to acidify in milk was preliminary tested to see if they could be used in cheesemaking with the dual function of starter and functional cultures. Strains SIM12 and SIS16 showed a fast-acidifying activity, reaching pH 4.0 in about 10 h., while strains Lh43 and 1734, which were very slow acidifiers, were discharged (S1 Fig). The immunomodulating capacity of the cheeses obtained with SIM12 and SIS16 (S2 Fig) was then tested. To this end, cheese samples were solubilized with Na-citrate to obtain cheese suspensions (CS), which were tested both untreated (TQ-CS, with a viable count of approx. 10^8^ CFU/ml, S1 Table) or after pasteurization (P-CS, 10^3^ CFU/ml, S1 Table). Results (expressed as % response normalized to LPS) showed that cheese samples, however treated and coming from cheeses fermented by both SIM12 and SIS16, decreased the production of the pro-inflammatory IL-12p40 and increased that of the anti-inflammatory IL-10 cytokines by total PBMC (Fig 3, panels a, b, d, e). The effect, which took place in both TQ-CS and P-CS samples, was dose-dependent, i.e., increased with increasing the % of added samples, as confirmed by the statistical significance of the anti-inflammatory index (Fig 3, panels c, f). The highest production of IL-10 was achieved with a 12,5% concentration of P-CS from cheeses made with SIS16, achieving a percentage of response to LPS of around 300 (Fig 3, panel e). It is interesting to underline the low production of cytokines in CNTR, i.e., milk coagulated by acidification at pH 5.3 with lactic acid, compared to milk fermented using *L*. *helveticus* strains.

## Discussion

In recent years, there has been a marked increase of studies showing that cheese may have a great potential to act as a carrier for the administration of functional bacteria [23–26]. However, several factors should be considered to demonstrate the actual functionality of strains when included in cheese. Among them are stress resistance to which micro-organisms are subjected during the various phases of cheese processing, their viability in the product, and the maintenance of the health-promoting characteristics during storage and until food is consumed consumption [27]. Not least, few data are available to distinguish the contribution to health effects related to microbial metabolism from those intrinsically related to the food itself [2, 28]. This study had three purposes: (i) to search for functional features in *L*. *helveticus* strains and test their ability to develop and maintain health benefits (in particular, the immunomodulatory capacity) in cheese; to this regard, we focused on some of the traits (e.g. folate accumulation, proteolytic, peptidase, and AO activities) most often involved in immunomodulatory effects; (ii) to distinguish between the direct immunomodulant effect of the cheese matrix and that related to the microbial growth; (iii) linked to the latter, to assess whether the immunomodulation effect was attributable to the cells (live or dead) trapped into cheese or to the products of their metabolism. Our attention was focused on *L*. *helveticus*, a species that, thanks to the ability to survive various environmental stresses such as high temperatures and osmotic pressure or low pH and oxygen concentration, is of increasing industrial importance as it is easily adaptable to fermentation conditions, thus facilitating its integration into new health promoting foods [6]. We selected cheese isolates to increase the probability of using strains already adapted to the dairy matrix, therefore more suitable to be successfully applied in cheesemaking trials.

Only strain SIM12 showed the ability to produce a moderate level (19.25 ng/ml) of folate. The ability to accumulate similar amounts (27 ng/ml) of folate by strains grown in chemically defined folate-free medium has been reported in *L*. *plantarum* [29]. The folate pathway is responsible for multiple functions, including energy (ATP) production, methylation reactions for DNA and protein synthesis, and the production of immunomodulatory molecules, inosine and adenosine, which play an important role in immune signaling and cytotoxicity [30]. Also, many peptides deriving from milk proteins and accumulated by highly proteolytic *L*. *helveticus* strains were shown to stimulate the host's immune system or display a marked antioxidant activity [5, 31]. The four studied strains showed a variable, although generally high, proteolytic and aminopeptidase activity in milk. In particular, our attention was focused on aminopeptidase activities typical of LAB isolated from cheese (leu-, arg-, lys-, and X prolyl dipeptidyl-aminopeptidase), therefore more likely to be involved in the release of (bioactive) peptides and free amino acids from milk proteins [32]. The AO activity, which was evaluated in milk culture supernatants using the ABTS, FTG, and FRAP assays, was also very variable. Notably, the literature currently reports a considerable variability of data due to the wide variety of poorly comparable analytical methods, which implies a difficulty in setting a threshold value for classifying strains with high or low AO activity [33]. However, the AO values for *L*. *helveticus* were low when compared with literature data on milks fermented by probiotic strains of *L*. *plantarum* [18, 34, 35]. Interestingly, strains SIS16 and Lh43, which expressed the highest FRAP and ABTS values, respectively, resulted also the most proteolytic ones. Finally, very high β-galactosidase activity was expressed from the tested *L*. *helveticus* strains. The evaluation of β-galactosidase (β-gal) activity had a dual purpose. Firstly, β-gal activity is an important functional feature. Lactose intolerance is found in people lacking β-gal which determines, in the upper regions of the small intestine, an inability to hydrolyze and assimilate this sugar, which is instead used from gas-producing microorganisms in the gut [36]. Secondly, a high β-gal activity allows LAB to produce lactic acid rapidly and reliably [37], thus facilitating their inclusion in starter cultures.

The immunomodulatory activity of the four strains and their cellular soluble postbiotics on moDCs, which are pivotal in the initiation of the adaptive immune response [8], was studied. To this regard, moDCs need to be activated by lipopolysaccharides (LPS) or pathogenic bacteria and, once activated, they produce cytokines [4]. Traditionally, IL10/IL12 ratio produced by moDCs is one of the main indexes for the classification of strains as potentially anti-inflammatory [4]. Therefore, we quantified the production of the anti-inflammatory IL-10 and the pro-inflammatory IL-12p70 by LPS- or *Salmonella*-stimulated moDCs, incubated in the presence of the four *L*. *helveticus* strains (or their postbiotics) [35]. In culture media, bacterial cells exerted a prevalent anti-inflammatory effect when inflammation was caused by LPS whereas, in the absence of stimulus, strains SIM12 and SIS16 strains induced an inflammatory response local that resembled that induced by *Salmonella*. Thus, SIM12 and SIS16 could be relevant under steady-state conditions to boost the immune response (immuno-enhancing effect) and increase resistance to pathogen infection or for vaccination purposes [8]. An immunomodulatory effect (i.e., an increase of the Il-10/IL12p70 ratio) on whatever induced inflammation was more marked using postbiotics of the four strains.

The immunomodulating activity was then tested in Caciotta-like cheese, which was chosen as a model cheese. Caciotta belongs to a large category of Italian traditional soft cheeses, is relatively easy to produce and has a very limited maturation period (www.assolatte.it). Cheeses were produced using individually the strains SIM12 and SIS16 which, differently from Lh43 and 1734, had the dual function of acidifying and immunomodulating cultures. This choice avoided to include an additional acidifying culture, which could have interfered with the immunomodulatory activity of *L*. *helveticus*. Data showed that immunomodulators could be present also in cheese, thus confirming tests in culture media. The substantial similarity of the anti-inflammatory index between TQ-CS (containing a high number of alive cells) and P-CS (where 99.999% of the viable cells were inactivated by pasteurization) suggested that the immunomodulation was essentially due to either a paraprobiotic effect, i.e. beyond cell viability, or soluble metabolites (postbiotics) accumulated by the *L*. *helveticus* strains through either cheese fermentation (e.g. folic acid, antioxidants, lactic acid) or substrate modification (e.g. proteolysis and peptide-lysis leading to the possible accumulation of bioactive, small peptides) or both [4, 5, 38]. Importantly, no cytokine production was induced by unfermented cheese, confirming that the immunomodulation was exclusively related to the presence and/or metabolic activity of strains SIM12 and SIS16. Several studies reported the ability of strains belonging to *L*. *helveticus* species to exert immunostimulatory effects, both when used alone or in combination with other bacterial species, but few are the reports on such effects in dairy products and, even less, in cheese [6]. In very early attempts, Laffineur *et al*. [39] demonstrated that the cell-free supernatant of *L*. *helveticus*-fermented ß-casein-enriched medium modulated *in vitro* the proliferation of lymphocytes. Ng and Griffiths [40] observed that cultured macrophages conditioned with *L*. *helveticus*-fermented milk, or its cell-free supernatant, were stimulated to secrete IL-6. Stuknyte *et al*. [41] observed a reduction of the immunomodulatory properties of casein hydrolysates when the bacterial cells of *L*. *helveticus* MIMLh5, used for the digestion of casein, were removed by ultrafiltration. More recently, a strain of *L*. *helveticus* isolated from fresh artisan Pasta Filata cheese showed *in vivo* anti-inflammatory effects as it stimulated the production of interleukin 10 (IL-10) and lowered that of the tumor necrosis factor alpha (TNF-α) by peripheral blood mononuclear cells (PBMC) [28]. This latter study and our results seem to stress that traditional dairy products should be more thoroughly and systematically considered as a natural source of health-promoting microorganisms [28]. In conclusion, this study adds further evidence on the potential benefits of *L*. *helveticus* for human health. Once validated in *in vivo* models, strains SIM12 and SIS16 could be good candidates for inclusion as functional cultures in cheese production.

## Supporting information

S1 Fig Acidification curves. Acidification curves of *Lactobacillus helveticus* strains SIM12, Lh43, SIS16, 1734 inoculated (1%) in microfiltered whole milk and incubated at 42°C for about 20 hours.
(TIF)

S2 Fig Cheese samples. Aspect of the cheese samples produced with *Lactobacillus helveticus* strains SIS16 and SIM12 respectively, after the 24 h storage at 4°C.
(TIF)

S1 Table Enumeration of viable cells in cheese suspensions. (DOCX)
