# Supplementary figures and images for: Functional characterization and immunomodulatory properties of Lactobacillus helveticus strains isolated from Italian hard cheeses
Source: PLoS One. 2021 Jan 25;16(1):e0245903. doi: 10.1371/journal.pone.0245903 (PMC7833162; doi:10.1371/journal.pone.0245903)

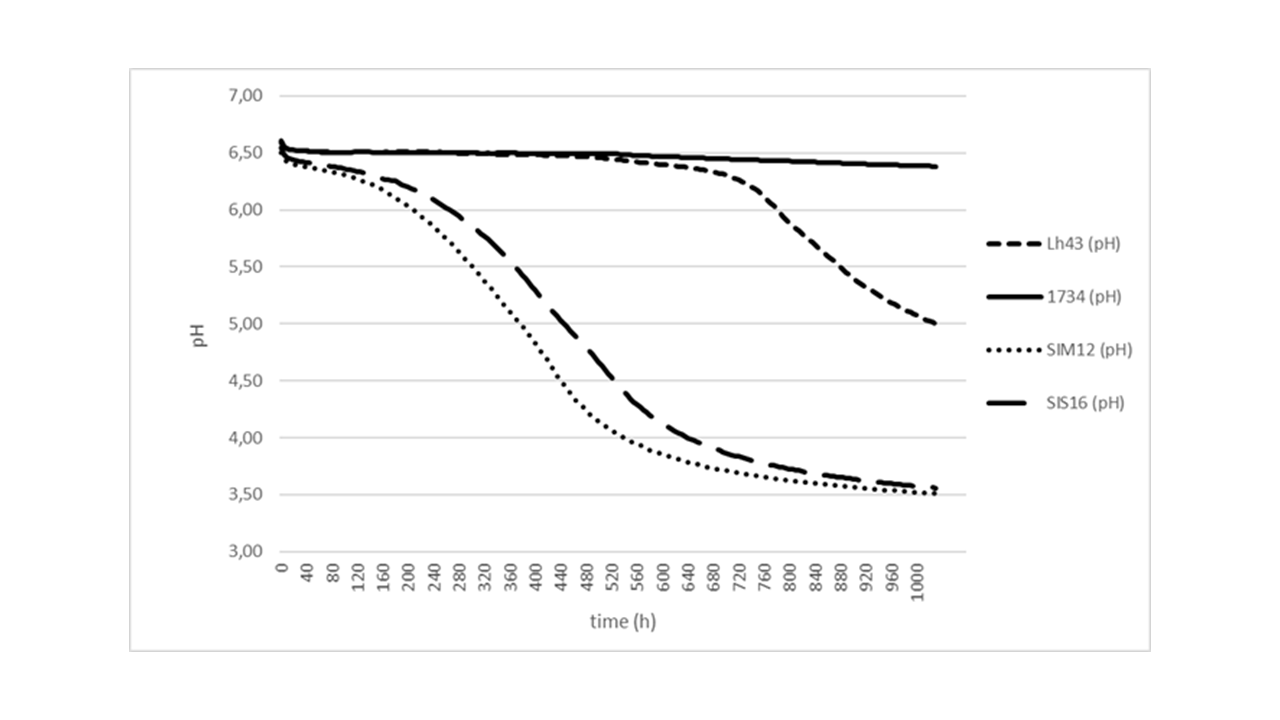

Supplement: S1 Fig — Acidification curves of Lactobacillus helveticus strains SIM12, Lh43, SIS16, 1734 inoculated (1%) in microfiltered whole milk and incubated at 42°C for about 20 hours. (TIF) [file pone.0245903.s001.tif]

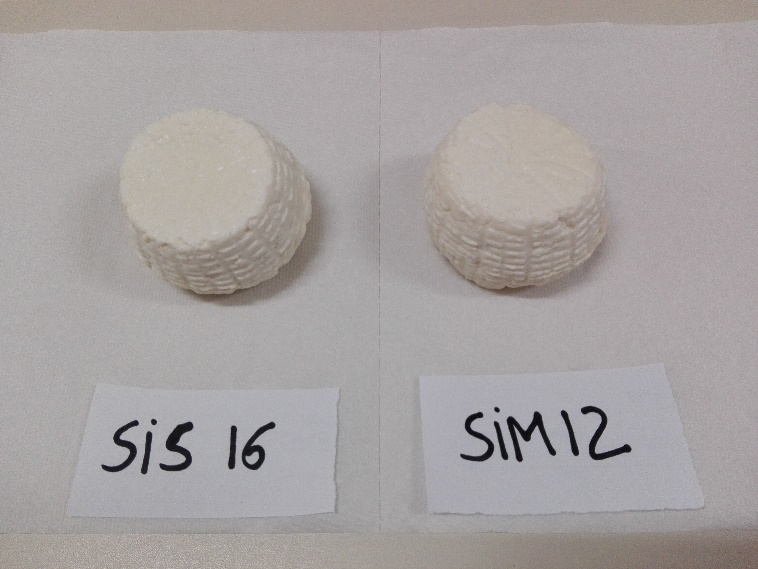

Supplement: S2 Fig — Aspect of the cheese samples produced with Lactobacillus helveticus strains SIS16 and SIM12 respectively, after the 24 h storage at 4°C. (TIF) [file pone.0245903.s002.tif]
