# Supplementary material for: Functional characterization and immunomodulatory properties of Lactobacillus helveticus strains isolated from Italian hard cheeses
Source: PLoS One. 2021 Jan 25;16(1):e0245903. doi: 10.1371/journal.pone.0245903 (PMC7833162; doi:10.1371/journal.pone.0245903)
Supplement: S1 Table — (DOCX) [file pone.0245903.s003.docx]

**S1 Table. Enumeration of viable cells in cheese suspensions**

| Samples | CFU/ml |
| --- | --- |
| TQ-CS with SIS16 | **1,75 x 10^8^** |
| P-CS with SIS 16 | **<10^3^** |
| TQ-CS with SIM12 | **6,00 x 10^8^** |
| P-CS with SIM12 | **5,50 x 10^3^** |

TQ-CS, cheese suspension directly used; P-CS, pasteurized cheese suspension.
